# Guidelines: The do’s, don’ts and don’t knows of direct observation of clinical skills in medical education

**DOI:** 10.1007/s40037-017-0376-7

**Published:** 2017-09-27

**Authors:** Jennifer R. Kogan, Rose Hatala, Karen E. Hauer, Eric Holmboe

**Affiliations:** 1 Perelman School of Medicine at the University of Pennsylvania, Philadelphia, PA USA; 2 University of British Columbia, Vancouver, British Columbia Canada; 3 University of California San Francisco, San Francisco, CA USA; 4 Accreditation Council of Graduate Medical Education, Chicago, IL USA

**Keywords:** Assessment, Clinical Skills, Competence, Direct Observation, Workplace Based Assessment

## Abstract

**Introduction:**

Direct observation of clinical skills is a key assessment strategy in competency-based medical education. The guidelines presented in this paper synthesize the literature on direct observation of clinical skills. The goal is to provide a practical list of Do’s, Don’ts and Don’t Knows about direct observation for supervisors who teach learners in the clinical setting and for educational leaders who are responsible for clinical training programs.

**Methods:**

We built consensus through an iterative approach in which each author, based on their medical education and research knowledge and expertise, independently developed a list of Do’s, Don’ts, and Don’t Knows about direct observation of clinical skills. Lists were compiled, discussed and revised. We then sought and compiled evidence to support each guideline and determine the strength of each guideline.

**Results:**

A final set of 33 Do’s, Don’ts and Don’t Knows is presented along with a summary of evidence for each guideline. Guidelines focus on two groups: individual supervisors and the educational leaders responsible for clinical training programs. Guidelines address recommendations for how to focus direct observation, select an assessment tool, promote high quality assessments, conduct rater training, and create a learning culture conducive to direct observation.

**Conclusions:**

High frequency, high quality direct observation of clinical skills can be challenging. These guidelines offer important evidence-based Do’s and Don’ts that can help improve the frequency and quality of direct observation. Improving direct observation requires focus not just on individual supervisors and their learners, but also on the organizations and cultures in which they work and train. Additional research to address the Don’t Knows can help educators realize the full potential of direct observation in competency-based education.

## Definitions of Do’s, Don’ts and Don’t Knows

**Do’s**—educational activity for which there is evidence of effectiveness

**Don’ts**—educational activity for which there is evidence of no effectiveness or of harms (negative effects)

**Don’t Knows**—educational activity for which there is no evidence of effectiveness

## Introduction

While direct observation of clinical skills is a key assessment strategy in competency-based medical education, it has always been essential to health professions education to ensure that all graduates are competent in essential domains [1, 2]. For the purposes of these guidelines, we use the following definition of competent: ‘Possessing the required abilities in all domains in a certain context at a defined stage of medical education or practice [1].’ Training programs and specialties have now defined required competencies, competency components, developmental milestones, performance levels and entrustable professional activities (EPAs) that can be observed and assessed. As a result, direct observation is an increasingly emphasized assessment method [3, 4] in which learners (medical students, graduate or postgraduate trainees) are observed by a supervisor while engaging in meaningful, authentic, realistic patient care and clinical activities [4, 5]. Direct observation is required by medical education accrediting bodies such as the Liaison Committee on Medical Education, the Accreditation Council of Graduate Medical Education and the UK Foundation Program [6–8]. However, despite its importance, direct observation of clinical skills is infrequent and the quality of observation may be poor [9–11]. Lack of high quality direct observation has significant implications for learning. From a formative perspective, learners do not receive feedback to support the development of their clinical skills. Also at stake is the summative assessment of learners’ competence and ultimately the quality of care provided to patients.

The guidelines proposed in this paper are based on a synthesis of the literature on direct observation of clinical skills and provide practical recommendations for both supervisors of learners and the educational leaders responsible for medical education clinical training programs. The objectives of this paper are to 1) help frontline teachers, learners and educational leaders improve the quality and frequency of direct observation; 2) share current perspectives about direct observation; and 3) identify gaps in understanding that could inform future research agendas to move the field forward.

## Methods

This is a narrative review [12] of the existing evidence coupled with the expert opinion of four medical educators from two countries who have research experience in direct observation and who have practical experience teaching, observing, and providing feedback to undergraduate (medical student) and graduate/postgraduate (resident/fellow) learners in the clinical setting. We developed the guidelines using an iterative process. We limited the paper’s scope to direct observation of learners interacting with patients and their families, particularly observation of history taking, physical exam, counselling and procedural skills. To create recommendations that promote and assure high quality direct observation, we focused on the frontline teachers/supervisors, learners, educational leaders, and the institutions that constitute the context. We addressed direct observation used for both formative and summative assessment. Although the stakes of assessment are a continuum, we define formative assessment as lower-stakes assessment where evidence about learner achievement is elicited, interpreted and used by teachers and learners to make decisions about next steps in instruction, while summative assessment is a higher-stakes assessment designed to evaluate the learner for the primary purpose of an administrative decision (i. e. progress or not, graduate or not, etc.) [13]. We excluded 1) observation of simulated encounters, video recorded encounters, and other skills (e. g. presentation skills, inter-professional team skills, etc.); 2) direct observation focused on practising physicians; and 3) other forms of workplace-based assessment (e. g. chart audit). Although an important aspect of direct observation is feedback to learners after observation, we agreed to limit the number of guidelines focused on feedback because a feedback guideline has already been published [14].

With these parameters defined, each author then independently generated a list of Do’s, Don’ts and Don’t Knows as defined below. We focused on Don’t Knows which, if answered, might change educational practice. Through a series of iterative discussions, the lists were reviewed, discussed and refined until we had agreed upon the list of Do’s, Don’ts and Don’t Knows. The items were then divided amongst the four authors; each author was responsible for identifying the evidence for and against assigned items. We primarily sought evidence explicitly focused on direct observation of clinical skills; however, where evidence was lacking, we also considered evidence associated with other assessment modalities. Summaries of evidence were then shared amongst all authors. We re-categorized items when needed based on evidence and moved any item for which there was conflicting evidence to the Don’t Know category. We used group consensus to determine the strength of evidence supporting each guideline using the indicators of strength from prior guidelines ([14]; Table 1). We did not give a guideline higher than moderate support when evidence came from extrapolation of assessment modalities other than direct observation.

**Table 1:** Criteria for strength of recommendation

Strong	A large and consistent body of evidence
Moderate	Solid empirical evidence from one or more papers plus consensus of the authors
Tentative	Limited empirical evidence plus the consensus of the authors

## Results

Our original lists had guidelines focused on three groups: individual supervisors, learners, and educational leaders responsible for training programs. This initial list of Do’s, Don’ts and Don’t Knows numbered 67 (35 Do’s, 16 Don’ts, 16 Don’t Knows). We reduced this to the 33 presented by combining similar and redundant items, with only two being dropped as unimportant based on group discussion. We decided to embed items focused on learners within the guidelines for educational leaders responsible for training programs to reduce redundancy and to emphasize how important it is for educational leaders to create a learning culture that activates learners to seek direct observation and incorporate feedback as part of their learning strategies.

After review of the evidence, four items originally defined as a Do were moved to a Don’t Know. The final list of Do’s, Don’ts and Don’t Knows is divided into two sections: guidelines that focus on individual supervisors (Table 2) and guidelines that focus on educational leaders responsible for training programs (Table 3). The remainder of this manuscript provides the key evidence to support each guideline and the strength of the guideline based on available literature.

**Table 2:** Summary of guidelines for direct observation of clinical skills for individual clinical supervisors

		**Strength of recommendation**
**Do’s**		
1.	Do observe authentic clinical work in actual clinical encounters	Strong
2.	Do prepare the learner prior to observation by discussing goals and setting expectations including the consequences and outcomes of the assessment	Strong
3.	Do cultivate learners’ skills in self-regulated learning	Moderate
4.	Do assess important clinical skills via direct observation rather than using proxy information	Strong
5.	Do observe without interrupting the encounter	Tentative
6.	Do recognize that cognitive bias, impression formation and implicit bias can influence inferences drawn during observation	Strong
7.	Do provide feedback after observation focusing on observable behaviours	Strong
8.	Do observe longitudinally to facilitate learners’ integration of feedback	Moderate
9.	Do recognize that many learners resist direct observation and be prepared with strategies to try to overcome their hesitation	Strong
**Don’ts**		
10.	Don’t limit feedback to quantitative ratings	Moderate
11.	Don’t give feedback in front of the patient without seeking permission from and preparing both the learner and the patient	Tentative
**Don’t Knows**		
12.	What is the impact of cognitive load during direct observation and what are approaches to mitigate it?	
13.	What is the optimal duration for direct observation of different clinical skills?

**Table 3:** Summary of guidelines for direct observation of clinical skills for educators/educational leaders

		**Strength of recommendation**
**Do’s**		
14.	Do select observers based on their relevant clinical skills and expertise	Strong
15.	Do use an assessment tool with existing validity evidence, when possible, rather than creating a new tool for direct observation	Strong
16.	Do train observers how to conduct direct observation, adopt a shared mental model and common standards for assessment, and provide feedback	Moderate
17.	Do ensure direct observation that aligns with program objectives and competencies (e. g. milestones)	Tentative
18.	Do establish a culture that invites learners to practice authentically and welcome feedback	Moderate
19.	Do pay attention to system factors that enable or inhibit direct observation	Moderate
**Don’ts**		
20.	Don’t assume that selecting the right tool for direct observation obviates the need for rater training	Moderate
21.	Don’t put the responsibility solely on the learner to ask for direct observation	Moderate
22.	Don’t underestimate faculty tension between being both a teacher and assessor	Tentative
23.	Don’t make all direct observations high-stakes; this will interfere with the learning culture around direct observation	Moderate
24.	When using direct observation for high-stakes summative decisions, don’t base decisions on too few direct observations by too few raters over too short a time and don’t rely on direct observation data alone	Strong
**Don’t Knows**		
25.	How do programs motivate learners to ask to be observed without undermining learners’ values of independence and efficiency?	
26.	How can specialties expand the focus of direct observation to important aspects of clinical practice valued by patients?	
27.	How can programs change a high-stakes, infrequent direct observation assessment culture to a low-stakes, formative, learner-centred culture?	
28.	What, if any, benefits are there to developing a small number of core faculty as ‘master educators’ who conduct direct observations?	
29.	Are entrustment-based scales the best available approach to achieve construct aligned scales, particularly for non-procedurally based specialties?	
30.	What are the best approaches to use technology to enable ‘on the fly’ recording of observational data?	
31.	What are the best faculty development approaches and implementation strategies to improve observation quality and learner feedback?	
32.	How should direct observation and feedback by patients or other members of the health care team be incorporated into direct observation approaches?	
33.	Does direct observation influence learner and patient outcomes?	

### Guidelines with supporting evidence for individual clinical supervisors doing direct observation

Do’s for individual supervisors

#### Guideline 1.

*Do observe authentic clinical work in actual clinical encounters.*

Direct observation, as an assessment that occurs in the workplace, supports the assessment of ‘does’ at the top of Miller’s pyramid for assessing clinical competence [15, 16]. Because the goal of training and assessment is to produce physicians who can practise in the clinical setting unsupervised, learners should be observed in the setting in which they need to demonstrate clinical competence. Actual clinical encounters are often more complex and nuanced than simulations or role plays and involve variable context; direct observation of actual clinical care enables observation of the clinical skills required to navigate this complexity [17].

Learners and teachers recognize that hands-on-learning via participation in clinical activities is central to learning [18–20]. Authenticity is a key aspect in contextual learning; the closer the learning is to real life, the more quickly and effectively skills can be learned [21, 22]. Learners also find real patient encounters and the setting in which they occur more natural, instructive and exciting than simulated encounters; they may prepare themselves more for real versus simulated encounters and express a stronger motivation for self-study [23]. Learners value the assessment and feedback that occurs after being observed participating in meaningful clinical care over time [24–26]. An example of an authentic encounter would be watching a learner take an initial history rather than watching the learner take a history on a patient from whom the clinical team had already obtained a history.

Although supervisors may try to observe learners in authentic situations, it is the authors’ experience that learners may default to inauthentic practice when being observed (for example, not typing in the electronic health record when taking a patient history or doing a comprehensive physical exam when a more focused exam is appropriate). While the impact of observer effects on performance is controversial (known as the Hawthorne effect) [11, 27], observers should encourage learners to ‘do what they would normally do’ so that learners can receive feedback on their actual work behaviours. Observers should not use fear of the Hawthorne effect as a reason not to observe learners in the clinical setting [see Guideline 18].

#### Guideline 2.

*Do prepare the learner prior to observation by discussing goals and setting expectations, including the consequences and outcomes of the assessment*.

Setting goals should involve a negotiation between the learner and supervisor and, where possible, direct observation should include a focus on what learners feel they most need. Learners’ goals motivate their choices about what activities to engage in and their approach to those activities. Goals oriented toward learning and improvement rather than performing well and ‘looking good’ better enable learners to embrace the feedback and teaching that can accompany direct observation [28, 29]. Learners’ autonomy to determine when and for what direct observation will be performed can enhance their motivation to be observed and shifts their focus from performance goals to learning goals [30, 31]. Teachers can foster this autonomy by soliciting learners’ goals and adapting the focus of their teaching and observation to address them. For example, within the same clinical encounter, a supervisor can increase the relevance of direct observation for the learner by allowing the learner to select the focus of observation and feedback-history taking, communication, or patient management. A learner’s goals should align with program objectives, competencies (e. g. milestones) and specific individual needs [see Guideline 17]. Asking learners at all levels to set goals helps normalize the importance of improvement for all learners rather than focusing on struggling learners. A collaborative approach between the observer and learner fosters the planning of learning, the first step in the self-regulated learning cycle described below [32]. Learners are receptive to being asked to identify and work towards specific personalized goals, and doing so instills accountability for their learning [31].

Prior to observation, observers should also discuss with the learner the consequences of the assessment. It is important to clarify when the observation is being used for feedback as opposed to high-stakes assessment. Learners often do not recognize the benefits of the formative learning opportunities afforded by direct observation, and hence explaining the benefits may be helpful [33].

#### Guideline 3.

*Do cultivate learners’ skills in self-regulated learning.*

**Fig. 1:**
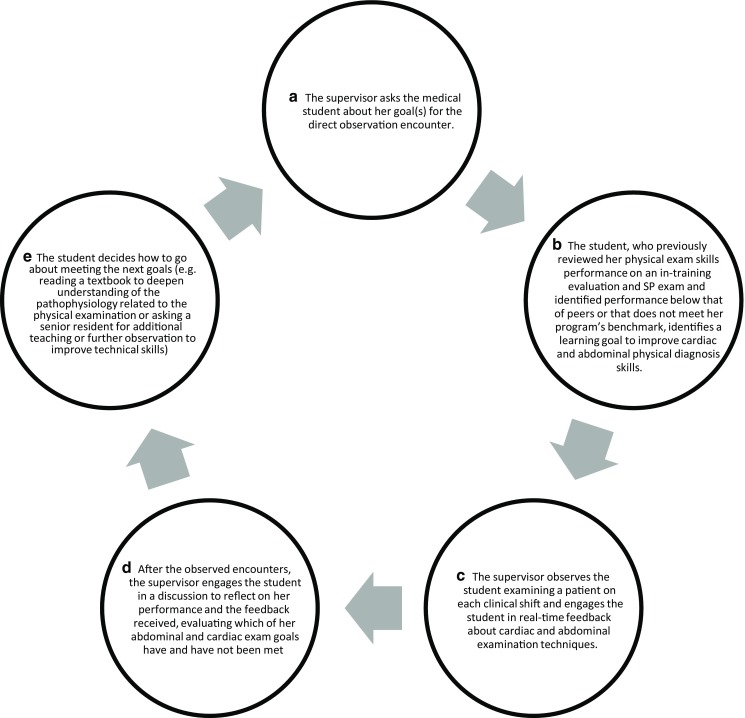
An example of using self-regulated learning in the context of direct observation. Self-regulated learning describes an ongoing cycle of (1) planning for one’s learning (*A,* *B,* *E*), (2) self-monitoring during an activity and making needed adjustments to optimize learning and performance (*C,* *D*), and (3) reflecting after an activity about whether a goal was achieved or where and why difficulties were encountered (*D,* *E*)

For direct observation to enhance learning, the learner should be prepared to use strategies that maximize the usefulness of feedback received to achieve individual goals. Awareness of one’s learning needs and actions needed to improve one’s knowledge and performance optimize the value of being directly observed. Self-regulated learning describes an ongoing cycle of 1) planning for one’s learning; 2) self-monitoring during an activity and making needed adjustments to optimize learning and performance; and 3) reflecting after an activity about whether a goal was achieved or where and why difficulties were encountered [32]. An example in the context of direct observation is shown in Fig. 1. Self-regulated learning is maximized with provision of small, specific amounts of feedback during an activity [34] as occurs in the context of direct observation. Trainees vary in the degree to which they augment their self-assessed performance by seeking feedback [35]. Direct observation combined with feedback can help overcome this challenge by increasing the amount of feedback learners receive [see Program Guideline 18].

#### Guideline 4.

*Do assess important clinical skills via direct observation rather than using proxy information*.

Supervisors should directly observe skills they will be asked to assess. In reality, supervisors often base their assessment of a learner’s clinical skills on proxy information. For example, supervisors often infer history and physical exam skills after listening to a learner present a patient or infer interpersonal skills with patients based on learner interactions with the team [36]. Direct observation improves the quality, meaningfulness, reliability and validity of clinical performance ratings [37]. Supervisors and learners consider assessment based on direct observation to be one of the most important characteristics of effective assessors [38]. Learners are also more likely to find in-training assessments valuable, accurate and credible when they are grounded in first-hand information of the trainee based on direct observation [39]. For example, if history taking is a skill that will be assessed at the end of a rotation, supervisors should directly observe a learner taking a history multiple times over the rotation.

#### Guideline 5.

*Do observe without interrupting the encounter.*

Observers should enable learners to conduct encounters uninterrupted whenever possible. Learners value autonomy and progressive independence [40, 41]. Many learners already feel that direct observation interferes with learning, their autonomy and their relationships with patients, and interruptions exacerbate these concerns [42, 43]. Interrupting learners as they are involved in patient care can lead to the omission of important information as shown in a study of supervisors who interrupted learners’ oral case presentations (an example of direct observation of clinical reasoning) [44]. Additionally, assessors often worry that their presence in the room might undermine the learner-patient relationship. Observers can minimize intrusion during direct observation by situating themselves in the patient’s peripheral vision so that the patient preferentially looks at the learner. This positioning should still allow the observer to see both the learner’s and patient’s faces to identify non-verbal cues. Observers can also minimize their presence by not interrupting the learner-patient interaction unless the learner makes egregious errors. Observers should avoid distracting interruptions such as excessive movement or noises (e. g. pen tapping).

#### Guideline 6.

*Do recognize that cognitive bias, impression formation and implicit bias can influence inferences drawn during observation*.

There are multiple threats to the validity of assessments derived from direct observation. Assessors develop immediate impressions from the moment they begin observing learners (often based on little information) and often feel they can make a performance judgment quickly (within a few minutes) [45, 46]. These quick judgments, or impressions, help individuals perceive, organize and integrate information about a person’s personality or behaviour [47]. Impression formation literature suggests that these initial judgments or inferences occur rapidly and unconsciously and can influence future interactions, what is remembered about a person and what is predicted about their future behaviours [48]. Furthermore, judgments about a learner’s competence may be influenced by relative comparisons to other learners (contrast effects) [49]. For example, a supervisor who observes a learner with poor skills and then observes a learner with marginal skills may have a more favourable impression of the learner with marginal skills than if they had previously observed a learner with excellent skills. Observers should be aware of these biases and observe long enough so that judgments are based on observed behaviours. Supervisors should focus on low inference, observable behaviours rather than high inference impressions. For example, if a learner is delivering bad news while standing up with crossed arms, the observable behaviour is that the learner is standing with crossed arms. The high inference impression is that this behaviour represents a lack of empathy or discomfort with the situation. Observers should not assume their high-level inference is accurate. Rather they should explore with the learner what crossed arms can mean as part of non-verbal communication [see Guideline 16].

#### Guideline 7.

*Do provide feedback after observation focusing on observable behaviours*.

Feedback after direct observation should follow previously published best practices [14]. Direct observation is more acceptable to learners when it is accompanied by timely, behaviourally based feedback associated with an action plan [50]. Feedback after direct observation is most meaningful when it addresses a learner’s immediate concerns, is specific and tangible, and offers information that helps the learner understand what needs to be done differently going forward to improve [31, 51]. Describing what the learner did well is important because positive feedback seems to improve learner confidence which, in turn, prompts the learner to seek more observation and feedback [31]. Feedback is most effective when it is given in person; supervisors should avoid simply documenting feedback on an assessment form without an in-person discussion.

#### Guideline 8.

*Do observe longitudinally to facilitate learners’ integration of feedback.*

Learning is facilitated by faculty observing a learner repeatedly over time, which also enables a better picture of professional development to emerge. Learners appreciate when they can reflect on their performance and, working in a longitudinal relationship, discuss learning goals and the achievement of those goals with a supervisor [25, 31]. Longitudinal relationships afford learners the opportunity to have someone witness their learning progression and provide feedback in the context of a broader view of them as a learner [25]. Ongoing observation can help supervisors assess a learner’s capabilities and limitations, thereby informing how much supervision the learner needs going forward [52]. Autonomy is reinforced when learners who are observed performing clinical activities with competence are granted the right to perform these activities with greater independence in subsequent encounters [53]. Experienced clinical teachers gain skill in tailoring their teaching to an individual learner’s goals and needs; direct observation is a critical component of this learner-centred approach to teaching and supervision [54]. If the same supervisor cannot observe a learner longitudinally, it is important that this sequence of observations occurs at the programmatic level by multiple faculty.

#### Guideline 9.

*Do recognize that many learners resist direct observation and be prepared with strategies to try to overcome their hesitation.*

Although some learners find direct observation useful [24, 55], many view it (largely independent of the assessment tool used) as a ‘tick-box exercise’ or a curricular obligation [33, 56]. Learners may resist direct observation for multiple reasons. They can find direct observation anxiety-provoking, uncomfortable, stressful and artificial [31, 43, 50, 57, 58]. Learners’ resistance may also stem from their belief that faculty are too busy to observe them [43] and that they will struggle to find faculty who have time to observe [59]. Many learners (correctly) believe direct observation has little educational value when it is only used for high-stakes assessments rather than feedback [60]; they do not find direct observation useful without feedback that includes teaching and planning for improvement [59]. One study audiotaped over a hundred feedback sessions as part of the mini-CEX and found faculty rarely helped to create an action plan with learners [61]. Learners perceive a conflict between direct observation as an educational tool and as an assessment method [57, 60]. Many learners feel that direct observation interferes with learning, autonomy, efficiency, and relationships with their patients [42, 43]. Furthermore, learners value handling difficult situations independently to promote their own learning [62, 63].

Supervisors can employ strategies to decrease learners’ resistance to direct observation. Learners are more likely to engage in the process of direct observation when they have a longitudinal relationship with the individual doing the observations. Learners are more receptive when they feel a supervisor is invested in them, respects them, and cares about their growth and development [31, 64]. Observation should occur frequently because learners generally become more comfortable with direct observation when it occurs regularly [58]. Discussing the role of direct observation for learning and skill development at the beginning of a rotation increases the amount of direct observation [43]. Supervisors should let learners know they are available for direct observation. Supervisors should make the stakes of the observation clear to learners, indicating when direct observation is being used for feedback and development versus for higher-stakes assessments. Supervisors should remember learners regard direct observation for formative purposes more positively than direct observation for summative assessment [65]. Additionally, learners are more likely to value and engage in direct observation when it focuses on their personalized learning goals [31] and when effective, high quality feedback follows.

Don’ts for individual supervisors

#### Guideline 10.

*Don’t limit feedback to quantitative ratings.*

Narrative comments from direct observations provide rich feedback to learners. When using an assessment form with numerical ratings, it is important to also provide learners with narrative feedback. Many direct observation assessment tools prompt evaluators to select numerical ratings to describe a learner’s performance [66]. However, meaningful interpretation of performance scores requires narrative comments that provide insight into raters’ reasoning. Narrative comments can support credible and defensible decision making about competence achievement [67]. Moreover, narrative feedback, if given in a constructive way, can help trainees accurately identify strengths and weaknesses in their performance and guide their competence development [46]. Though evidence is lacking in direct observation per se and quantitative ratings are not the same as grades, other assessment literature suggests that learners do not show learning gains when they receive just grades or grades with comments. It is hypothesized that learning gains do not occur when students receive grades with comments because learners focus on the grade and ignore the comments [68–70]. In contrast, learners who receive only comments (without grades) show large learning gains [68–70]. Grades without narrative feedback fail to provide learners with sufficient information and motivation to stimulate improvement [26]. The use of an overall rating may also reduce acceptance of feedback [51] although a Pass/Fail rating may be better received by students than a specific numerical rating [71]. Although the pros and cons of sharing a rating with a learner after direct observation are not known, it is important that learners receive narrative feedback that describes areas of strength (skills performed well) and skills requiring improvement when direct observation is being used for formative assessment.

#### Guideline 11.

*Don’t give feedback in front of the patient without seeking permission from and preparing both the learner and the patient*.

If a supervisor plans to provide feedback to a learner after direct observation in front of a patient, it is important to seek the learner’s and patient’s permission in advance. This permission is particularly important since feedback is typically given in a quiet, private place, and feedback given in front of the patient may undermine the learner-patient relationship. If permission has not been sought or granted, the learner should not receive feedback in front of the patient. The exception, however, is when a patient is not getting safe, effective, patient-centred care; in this situation, immediate interruption is warranted (in a manner that supports and does not belittle the learner), recognizing that this interruption is a form of feedback.

Although bedside teaching can be effective and engaging for learners, [72, 73] some learners feel that teaching in front of the patient undermines the patient’s therapeutic alliance with them, creates a tense atmosphere, and limits the ability to ask questions [73, 74]. However, in the era of patient-centredness, the role and importance of the patient voice in feedback may increase. In fact, older studies suggest many patients want the team at the bedside when discussing their care [75]. How to best create a therapeutic and educational alliance with patients in the context of direct observation requires additional attention.

Don’t Knows for individual supervisors

#### Guideline 12.

*What is the impact of cognitive load during direct observation and what are approaches to mitigate it?*

An assessor can experience substantial cognitive load observing and assessing a learner while simultaneously trying to diagnose and care for the patient [76]. Perceptual load may overwhelm or exceed the observer’s attentional capacities. This overload can cause ‘inattentional blindness,’ where focusing on one stimulus impairs perception of other stimuli [76]. For example, focusing on a learner’s clinical reasoning while simultaneously trying to diagnose the patient may interfere with the supervisor’s ability to attend to the learner’s communication skills. As the number of dimensions raters are asked to assess increases, the quality of ratings decreases [77]. More experienced observers develop heuristics, schemas or performance scripts about learners and patients to process information and thereby increase observational capacity [45, 76]. More highly skilled faculty may also be able to detect strengths and weaknesses with reduced cognitive load because of the reduced effort associated with more robust schemes and scripts [78]. Assessment instrument design may also influence cognitive load. For example, Byrne and colleagues, using a validated instrument to measure cognitive load, showed that faculty experienced greater cognitive load when they were asked to complete a 20 plus item checklist versus a subjective rating scale for an objective structured clinical examination of a trainee inducing anaesthesia [79]. More research is needed to determine the impact of cognitive load during direct observation in non-simulated encounters and how to structure assessment forms so that observers are only asked to assess critical elements, thereby limiting the number of items to be rated.

#### Guideline 13.

*What is the optimal duration for direct observation of different skills?*

Much of the recent direct observation and feedback literature has focused on keeping direct observation short and focused to promote efficiency in a busy workplace [80]. While short observations make sense for clinical specialties that have short patient encounters, for other specialties relevant aspects of practice that are only apparent with a longer observation may be missed with brief observations. One of the pressing questions for direct observation and feedback is to determine the optimal duration of encounters for various specialties, learners and skills. The optimal duration of an encounter will likely need to reflect multiple variables including the patient’s needs, the task being observed, the learner’s competence and the faculty’s familiarity with the task [78, 81].

### Guidelines with supporting evidence for educators/educational leaders

Do’s for educational leaders

#### Guideline 14.

*Do select observers based on their relevant clinical skills and expertise.*

Educational leaders, such as program directors, should select observers based on their relevant clinical skills and educational expertise. Content expertise (knowledge of what exemplar skill looks like and having the ability to assess it) is a prerequisite for fair, credible assessment [82]. However, assessors are often asked to directly observe skills for which they feel they lack content expertise, and assessors do not believe using a checklist can make up for a lack of their own clinical skill [83]. Additionally, a supervisor’s own clinical skills may influence how they assess a learner [78]. When assessors’ idiosyncrasy is the result of deficiencies in their own competencies [84] and when assessors use themselves as the gold standard during observation and feedback, learners may acquire the same deficiencies or dyscompetencies [78, 85–90]. Because faculty often use themselves as the standard by which they assess learner performance (i. e. frame of reference), [82] it is important to select assessors based on their clinical skills expertise or provide assessor training so assessors can recognize competent and expert performance without using themselves as a frame of reference.

At a programmatic level, it is prudent to align the types of observations needed to individuals who have the expertise to assess that particular skill. For example, a program director might ask cardiologists to observe learners’ cardiac exams and ask palliative care physicians to observe learners’ goals of care discussions. Using assessors with content expertise and clinical acumen in the specific skill(s) being assessed is also important because learners are more likely to find feedback from these individuals credible and trustworthy [20, 64]. When expertise is lacking, it is important to help faculty correct their dyscompetency [91]. Faculty development around assessment can theoretically become a ‘two-for-one’—improving the faculty’s own clinical skills while concomitantly improving their observation skills [91].

In addition to clinical skills expertise, assessors also must have knowledge of what to expect of learners at different training levels [83]. Assessors must be committed to teaching and education, invested in promoting learner growth, interested in learners’ broader identity and experience, and willing to trust, respect and care for learners [64] [see Guideline 28].

#### Guideline 15.

*Do use an assessment tool with existing validity evidence, when possible, rather than creating a new tool for direct observation.*

Many tools exist to guide the assessment of learners’ performance based on direct observation [66, 92]. Rather than creating new tools, educators should, when possible, use existing tools for which validity evidence exists [93]. When a tool does not exist for an educator’s purpose, options are to adapt an existing tool or create a new one. Creating a new tool or modifying an existing tool for direct observation should entail following guidelines for instrument design and evaluation, including accumulating validity evidence [94]. The amount of validity evidence needed will be greater for tools used for high-stakes summative assessments than for lower-stakes formative assessments.

Tool design can help optimize the reliability of raters’ responses. The anchors or response options on a tool can provide some guidance about how to rate a performance; for example, behavioural anchors or anchors defined as milestones that describe the behaviour along a spectrum of developmental performance can improve rater consistency [95]. Scales that query the supervisor’s impressions about the degree of supervision the learner needs or the degree of trust the supervisor feels may align better with how supervisors think [96]. A global impression may better capture performance reliably across raters than a longer checklist [97]. The choice between the spectrum of specific checklists to global impressions, and everything in between, depends primarily on the purpose of the assessment. For example, if feedback is a primary goal, holistic ratings possess little utility if learners do not receive granular, specific feedback. Regardless of the tool selected, it is important for tools to provide ample space for narrative comments [71] [see Guideline 16 and 30].

Importantly, validity ultimately resides in the user of the instrument and the context in which the instrument is used. One could argue that the assessors (e. g. faculty), in direct observation, *are* the instrument. Therefore, program directors should recognize that too much time is often spent designing tools rather than training the observers who will use them [see Guideline 20].

#### Guideline 16.

*Do train observers how to conduct direct observation, adopt a shared mental model and common standards for assessment, and provide feedback*.

The assessments supervisors make after observing learners with patients are highly variable, and supervisors observing the same encounter assess and rate the encounter differently regardless of the tool used. Variability results from observers focusing on and prioritizing different aspects of performance and applying different criteria to judge performance [46, 82, 98, 99]. Assessors also use different definitions of competence [82, 98]. The criteria observers use to judge performance are often experientially and idiosyncratically derived, are commonly influenced by recent experiences, [49, 82, 100] and can be heavily based on first impressions [48]. Assessors develop idiosyncrasies as a result of their own training and years of their own clinical and teaching practices. Such idiosyncrasies are not necessarily unhelpful if based on strong clinical evidence and best practices. For example, an assessor may be an expert in patient-centred interviewing and heavily emphasize such behaviours and skills during observation to the exclusion of other aspects of the encounter [47]. While identifying outstanding or very weak performance is considered straightforward, decisions about performance in ‘the grey area’ are more challenging [83].

Rater training can help overcome but not eliminate these limitations of direct observation. Performance dimension training is a rater training approach in which participants come to a shared understanding of the aspects of performance being observed and criteria for rating performance [9]. For example, supervisors might discuss what are the important skills when counselling a patient about starting a medication. Most assessors welcome a framework to serve as a scaffold or backbone for their judgments [83]. Supervisors who have done performance dimension training describe how the process provides them with a shared mental model about assessment criteria that enables them to make more standardized, systematic, comprehensive, specific observations, pay attention to skills they previously did not attend to, and improve their self-efficacy giving specific feedback [91].

Frame of reference training builds upon performance dimension training by teaching raters to use a common conceptualization (i. e., frame of reference) of performance during observation and assessment by providing raters with appropriate standards pertaining to the rated dimensions [101]. A systematic review and meta-analysis from the non-medical performance appraisal literature demonstrated that frame of reference training significantly improved rating accuracy with a moderate effect size [101, 102]. In medicine, Holmboe et al. showed that an 8‑hour frame of reference training session that included live practice with standardized residents and patients modestly reduced leniency and improved accuracy in direct observation 8 months after the intervention [9]. However, brief rater training (e. g. half day workshop) has not been shown to improve inter-rater reliability [103].

Directors of faculty development programs should plan how to ensure that participants apply the rater training in their educational and clinical work. Strategies include making the material relevant to participants’ perceived needs and the format applicable within their work context [82, 104]. Key features of effective faculty development for teaching effectiveness also include the use of experiential learning, provision of feedback, effective peer and colleague relationships, intentional community building and longitudinal program design [105, 106]. Faculty development that focuses on developing communities of practice, a cadre of educators who look to each other for peer review and collaboration, is particularly important in rater training and is received positively by participants [91]. Group training highlights the importance of moving the emphasis of faculty development away from the individual to a community of educators invested in direct observation and feedback. Because assessors experience tension giving feedback after direct observation, particularly when it comes to giving constructive feedback [107], assessor training should also incorporate teaching on giving effective feedback after direct observation [see Guideline 32]. While rater training is important, a number of unanswered questions about rater training still remain [see Guideline 31].

#### Guideline 17.

*Do ensure direct observation aligns with program objectives and competencies (e. g. milestones).*

Clearly articulated program goals and objectives set the stage for defining the purposes of direct observation [93]. A defined framework for assessment aligns learners’ and supervisors’ understandings of educational goals and guides selection of tools to use for assessment. Program directors may define goals and objectives using an analytic approach (‘to break apart’) defining the components of practice to be observed, from which detailed checklists can be created [108]. A synthetic approach can also be used to define the work activities required for competent, trustworthy practice, from which more holistic scales such as ratings of entrustment can be applied [109]. Program directors can encourage supervisors and learners to refer to program objectives, competencies, milestones and EPAs used in the program when discussing learner goals for direct observation.

#### Guideline 18.

*Do establish a culture that invites learners to practice authentically and welcome feedback.*

Most learners enter medical school from either pre-university or undergraduate cultures heavily steeped in grades and high-stakes tests. In medical school, grades and tests can still drive substantial learner behaviour, and learners may still perceive low-stakes assessments as summative obstacles to be surmounted rather than as learning opportunities. Learners often detect multiple conflicting messages about expectations for learning or performance [60, 110]. How then can the current situation be changed to be more learner centred?

Programs should explicitly identify for learners when and where the learning culture offers low-stakes opportunities for practice, and programs should foster a culture that enables learners to embrace direct observation as a learning activity. In the clinical training environment, learners need opportunities to be observed performing the skills addressed in their learning goals by supervisors who minimize perceived pressures to appear competent and earn high marks [111]. Orientations to learning (learner or mastery goals versus performance goals) influence learning outcomes [112]. A mastery-oriented learner strives to learn, invites feedback, embraces challenges, and celebrates improvement. Conversely, a performance-oriented learner seeks opportunities to appear competent and avoid failure. A culture that enables practice and re-attempting the same skill or task, and rewards effort and improvement, promotes a mastery orientation. A culture that emphasizes grades, perfection or being correct at the expense of learning can promote maladaptive ‘performance-avoid’ goals in which learners actively work to avoid failure, as in avoiding being directly observed [28]. Programs should encourage direct observers to use communication practices that signal the value placed on practice and effort rather than just on correctness. Separating the role of teacher who conducts direct observation and feedback in low-stakes settings from the role of assessor makes these distinctions explicit for learners. Programs should also ensure their culture promotes learner receptivity to feedback by giving learners personal agency over their learning and ensuring longitudinal relationships between learners and their supervisors [14, 26].

#### Guideline 19.

*Do pay attention to systems factors that enable or inhibit direct observation.*

The structure and culture of the medical training environment can support the value placed on direct observation. Trainees pay attention to when and for what activities their supervisors observe them and infer, based on this, which educational and clinical activities are valued [42].

A focus on patient-centred care within a training environment embeds teaching in routine clinical care through clinical practice tasks shared between learners and supervisors within microsystems [113]. Faculty buy-in to the process of direct observation can be earned through education about the importance of direct observation for learning and through schedule structures that enable faculty time with learners at the bedside [93]. Training faculty to conduct direct observation as they conduct patient care frames this task as integral to efficient, high quality care and education [114]. Patient and family preferences for this educational strategy suggest that they perceive it as beneficial to their care, and clinicians can enjoy greater patient satisfaction as a result [115, 116].

Educational leaders must address the systems barriers that limit direct observation. Lack of time for direct observation and feedback is one of the most common barriers to direct observation. Programs need to ensure that educational and patient care systems (e. g. supervisor:learner ratios, patient census) allow time for direct observation and feedback. Attention to the larger environment of clinical care at teaching hospitals can uncover additional barriers that should be addressed to facilitate direct observation. The current training environment is too often characterized by a fast-paced focus on completing work at computers using the electronic health record, with a minority of trainee time spent interacting with patients or in educational activities [117, 118]. Not uncommonly, frequent shifts in supervisor-learner pairings make it difficult for learners to be observed by the same supervisor over time in order to incorporate feedback and demonstrate improvement [119]. Program directors should consider curricular structures that afford longitudinal relationships that enhance supervisors’ and learners’ perceptions of the learning environment, the ability to give and receive constructive feedback and trust the fairness of judgments about learners [26, 120, 121]. Redesign of the ambulatory continuity experience in graduate medical education shows promise to foster these longitudinal opportunities for direct observation and feedback [122].

Don’ts focused on program

#### Guideline 20.

*Don’t assume that selecting the right tool for direct observation obviates the need for rater training.*

Users of tools for direct observation may erroneously assume that a well-designed tool will be clear enough to raters that they will all understand how to use it. However, as described previously, regardless of the tool selected, observers should be trained to conduct direct observations and record their observations using the tool. The actual measurement instrument is the faculty supervisor, not the tool.

#### Guideline 21.

*Don’t put the responsibility solely on the learner to ask for direct observation.*

Learners and their supervisors should together take responsibility for ensuring that direct observation and feedback occur. While learners desire meaningful feedback focused on authentic clinical performance, [31, 39] they commonly experience tension between valuing direct observation as useful to learning and wanting to be autonomous and efficient [42]. Changing the educational culture to one where direct observation is a customary part of daily activities, with acknowledgement of the simultaneous goals of direct observation, autonomy and efficiency, may ease the burden on learners. Removing or reducing responsibility from the learner to ask for direct observation and making it, in part, the responsibility of faculty and the program will promote shared accountability for this learning activity.

#### Guideline 22.

*Don’t underestimate faculty tension between being both a teacher and assessor.*

Two decades ago Michael J. Gordon described the conflict faculty experience being both teacher (providing guidance to the learner) and high-stakes assessor (reporting to the training program if the learner is meeting performance standards) [123]. In the current era of competency-based medical education, with increased requirements on faculty to report direct observation encounters, this tension persists. Gordon’s solution mirrors many of the developments of competency-based medical education: develop two systems, one that is learner-oriented to provide learners with feedback and guidance by the frontline faculty, and one that is faculty-oriented, to monitor or screen for learners not maintaining minimal competence, and for whom further decision making and assessment would be passed to a professional standards committee [124, 125]. Programs will need to be sensitive to the duality of faculty’s position when using direct observation in competency-based medical education and consider paradigms that minimize this role conflict [126, 127].

#### Guideline 23.

*Don’t make all direct observations high stakes; this will interfere with the learning culture around direct observation.*

Most direct observations should not be high stakes, but rather serve as low-stakes assessments of authentic patient-centred care that enable faculty to provide guidance on the learner’s daily work. One of the benefits of direct observation is the opportunity to see how learners approach their authentic clinical work, but the benefits may be offset if the act of observation alters performance [128]. Learners are acutely sensitive to the ‘stakes’ involved in observation to the point that their performance is altered [11, 31]. In a qualitative study of residents’ perceptions of direct observation, residents reported changing their clinical style to please the observer and because they assumed the performance was being graded. The direct observation shifted the learner’s goals from patient-centred care to performance-centred care [11]. In another study, residents perceived that the absence of any ‘stakes’ of the direct observation (the conversations around the observations remained solely between observer and learner) facilitated the authenticity of their clinical performance [31].

#### Guideline 24.

*When using direct observation for high-stakes summative decisions, don’t base decisions on too few direct observations by too few raters over too short a time and don’t rely on direct observation data alone.*

A single assessment of a single clinical performance has well-described limitations: 1) the assessment captures the impression of only a single rater and 2) clinical performance is limited to a single content area whereas learners will perform differentially depending on the content, patient, and context of the assessment [129, 130]. To improve the generalizability of assessments, it is important to increase the number of raters observing the learner’s performance across a spectrum of content (i. e. diagnoses and skills) and contexts [131, 132]. Furthermore, a learner’s clinical performance at any given moment may be influenced by external factors such as their emotional state, motivation, or fatigue. Thus, capturing observations over a period of time allows a more stable measure of performance.

Combining information gathered from multiple assessment tools (e. g. tests of medical knowledge, simulated encounters, direct observations) in a program of assessment will provide a more well-rounded evaluation of the learner’s competence than any single assessment tool [133, 134]. Competence is multidimensional, and no single assessment tool can assess all dimensions in one format [130, 135]. This is apparent when examining the validity arguments supporting assessment tools; for any single assessment tool, there are always strengths and weaknesses in the argument [136, 137]. It is important to carefully choose the tools that provide the best evidence to aid decisions regarding competence [136, 137]. For example, if the goal is to assess a technical skill, a program may combine a knowledge test of the indications, contraindications and complications of the procedure with direct observation using part-task trainers in the simulation laboratory with direct observation in the real clinical setting (where the learner’s technical skills can be assessed in addition to their communication with the patient).

Don’t Knows

#### Guideline 25.

*How do programs motivate learners to ask to be observed without undermining learners’ values of independence and efficiency?*

While the difficulties in having a learning culture that simultaneously values direct observation/feedback and autonomy/efficiency have been discussed, solutions to this problem are less clear. Potential approaches might be to target faculty to encourage direct observation of short encounters (thus minimizing the impact on efficiency) and to ground the direct observation in the faculty’s daily work [66, 129]. However, these strategies can have the unintended effect of focusing direct observation on specific tasks which are amenable to short observations as opposed to competencies that require more time for direct observation (e. g. professional behaviour, collaboration skills etc.) [60]. Another solution may be to make direct observation part of daily work, such as patient rounds, hand-offs, discharge from a hospital or outpatient facility, clinic precepting and so forth. Leveraging existing activities reduces the burden of learners having to ask for direct observation, as occurs in some specialties and programs. For example, McMaster’s emergency medicine residency program has a system that capitalizes on supervisors’ direct observation during each emergency department shift by formalizing the domains for observation [138]. It will be important to identify additional approaches that motivate learners to ask for observation.

#### Guideline 26.

*How can specialties expand the focus of direct observation to important aspects of clinical practice valued by patients?*

Patients and physicians disagree about the relative importance of aspects of clinical care; for example, patients more strongly rate the importance of effective communication of health-related information [139]. If assessors only observe what they most value or are most comfortable with, how can the focus of direct observation be expanded to all important aspects of clinical practice [42]? In competency-based medical education, programs may take advantage of rotations that emphasize specific skills to expand the focus of direct observation to less frequently observed domains (e. g. using a rheumatology rotation to directly observe learners’ musculoskeletal exam skills as opposed to trying to observe joint exams on a general medicine inpatient service). What seems apparent is that expecting everyone to observe everything is an approach that has failed. Research is needed to learn how to expand the focus of direct observation for each specialty to encompass aspects of clinical care valued by patients. Many faculty development programs target only the individual faculty member, and relatively few target processes within organizations or cultural change [106]. As such, faculty development for educational leaders that target these types of process change is likely needed.

#### Guideline 27.

*How can programs change a high-stakes, infrequent direct observation assessment culture to a low-stakes, formative, learner-centred culture?*

The importance of focusing direct observation on formative assessment has been described. However, additional approaches are still needed that help change a high-stakes, infrequent direct observation assessment culture to a low-stakes, formative, learner-centred direct observation culture [140]. Studies should explore strategies for and impacts of increasing assessment frequency, empowering learners to seek assessment, [141, 142] and emphasizing to learners that assessment for feedback and coaching are important [143–145]. How a program effectively involves its learners in designing, monitoring and providing ongoing improvement of the assessment program also merits additional study [146].

Although direct observation should be focused on formative assessment, ultimately all training programs must make a high-stakes decision regarding promotion and transition. Research has shown that the more accurate assessment information a program has, the more accurate and better informed the high-stakes decisions are [134, 135]. Other than using multiple observations to make a high-stakes decision [140], it is not clear exactly how programs can best use multiple low-stakes direct observation assessments to make high-stakes decisions. Additionally, how do programs ensure that assessments are perceived as low stakes (i. e. that no one low-stakes observation will drive a high-stakes decision) when assessments ultimately will be aggregated for higher-stakes decisions?

Group process is an emerging essential component of programmatic assessment. In some countries these groups, called clinical competency committees, are now a required component of graduate medical education [147]. Robust data from direct observations, both qualitative and quantitative, can be highly useful for the group judgmental process to improve decisions about progression [148, 149]. While direct observation should serve as a critical input into a program of assessment that may use group process to enhance decision making regarding competence and entrustment, how to best aggregate data is still unclear.

#### Guideline 28.

*What, if any, benefits are there to developing a small number of core faculty as ‘master educators’ who conduct direct observations?*

One potential solution to the lack of direct observation in many programs may be to develop a parallel system with a core group of assessors whose primary role is to conduct direct observations without simultaneous responsibility for patient care. In a novel feedback program where faculty were supported with training and remuneration for their direct observations, residents benefited in terms of their clinical skills, development as learners and emotional well-being [31]. Such an approach would allow faculty development efforts to focus on a smaller cadre of educators who would develop skills in direct observation and feedback. A cadre of such educators would likely provide more specific and tailored feedback and their observations would complement the insights of the daily clinical supervisors, thus potentially enhancing learners’ educational experience [150]. Such an approach might also provide a work-around to the time constraints and busyness of the daily clinical supervisors. The structure, benefits and costs of this approach requires study.

#### Guideline 29.

*Are entrustment-based scales the best available approach to achieve construct aligned scales, particularly for non-procedurally-based specialties?*

While the verdict is still out, there is growing research that entrustment scales may be better than older scales that use adjectival anchors such as unsatisfactory to superior or poor to excellent [151, 152]. These older scales are examples of ‘construct misaligned scales’ that do not provide faculty with meaningful definitions along the scale and whether learner performance is to be compared with other learners or another standard. Construct aligned scales use anchors with or without narrative descriptors that ‘align’ with the educational mental construct of the faculty. Entrustment, often based on level of supervision, is better aligned with the type of decisions a faculty member has to make about a learner (e. g. to trust or not trust for reactive supervision). Crossley and colleagues, using a developmental descriptive scale on the mini-clinical evaluation exercise grounded in the Foundation years for UK trainees, found better reliability and acceptability using the aligned scale than the traditional mini-CEX [96]. Regehr and colleagues found asking faculty to use standardized, descriptive narratives versus typical scales led to better discrimination of performance among a group of residents [153]. Other investigators have also found better reliability and acceptability for observation instruments that use supervision levels as scale anchors [152, 154]. Thus entrustment scales appear to be a promising development though caution is still needed. Reliability is only one aspect of validity, and the same problems that apply to other tools can still be a problem with entrustment scales. For example, assessors can possess very different views of supervision (i. e. lack a shared mental model). Teman and colleagues found that faculty vary in how they supervise residents in the operating room; they argued that faculty development is needed to best determine what type of supervision a resident needs [155, 156]. The procedural Zwisch scale provides robust descriptors of supervisor behaviours that correlate with different levels of supervision [108, 154, 158]. Studies using entrustment scales have largely focused on procedural specialties; more research is needed to understand their utility in more non-procedural based specialties. Research is also needed to determine the relative merits of behaviourally anchored scales (focused on what a learner does) versus entrustment scales.

#### Guideline 30.

*What are the best approaches to use technology to enable ‘on the fly’ recording of observational data?*

Technology can facilitate the immediate recording of observational data such as ratings or qualitative comments. Much of the empirical medical education research on direct observation has focused on the assessment tool more than the format in which the tool is delivered [66]. However, given the evolution of clinical care from paper-based to electronic platforms, it makes intuitive sense that the recording, completion and submission of direct observations may be facilitated by using handheld devices or other electronic platforms. The few studies done in this realm have documented the feasibility of and user satisfaction with an electronic approach, but more research is necessary to understand how to optimize electronic platforms both to promote the development of shared goals, support observation quality and collect and synthesize observations [159–162].

#### Guideline 31.

*What are the best faculty development approaches and implementation strategies to improve observation quality and learner feedback?*

As already described, recent research in rater cognition provides some insights on key factors that affect direct observation: assessor idiosyncrasy, variable frames of reference, cognitive bias, implicit bias and impression formation [46, 47, 82, 98, 99]. However, how this can inform approaches to faculty development is not well understood. For example, what would be the value or impact of educational leaders helping assessors recognize their idiosyncratic tendency and ensuring learners receive sufficient longitudinal sampling from a variety of assessors to ensure all aspects of key competencies are observed? Would assessor idiosyncrasy and cognitive bias (e. g. contrast effect) be reduced by having assessors develop robust shared criterion-based mental models or use entrustment-based scales [96, 151, 152, 157]? [See Guideline 16].

While more intensive rater training based on the principles of performance dimension training and frame of reference training decreases rater leniency, improves rating accuracy, and improves self-assessed comfort with direct observation and feedback [9, 91], studies have not specifically explored whether rater training improves the quality of observation, assessment or feedback to learners.

The optimal structure and duration of assessor training is also unclear [9, 103, 157]. Direct observation is a complex skill and likely requires ongoing, not just one-time, training and practice. However, studies are needed to determine what rater training structures are most effective to improve the quality of direct observation, assessment and feedback. Just how long does initial training need to be? What type of longitudinal training or skill refreshing is needed? How often should it occur? What is the benefit of providing assessors feedback on the quality of their ratings or their narratives? Given that existing studies show full day training has only modest effects, it will be important to determine the feasibility of intensive training.

#### Guideline 32.

*How should direct observation and feedback by patients or other members of the health care team be incorporated into direct observation approaches?*

Patients and other health professionals routinely observe various aspects of learner performance and can provide feedback that complements supervisors’ feedback. It is very hard to teach and assess patient-centred care without involving the perspective and experiences of the patient. Given the importance of inter-professional teamwork, the same can be said regarding assessments from other team members. Patient experience surveys and multi-source feedback instruments (which may include a patient survey) are now commonly used to capture the observations and experiences of patients and health professionals [163, 164]. Multisource feedback, when implemented properly, can be effective in providing useful information and changing behaviour [165, 166]. What is not known is whether just-in-time feedback from patients would be helpful. Concato and Feinstein showed that asking the patient three questions at the end of the visit yielded rich feedback for the clinic and the individual physicians [167]. A patient-centred technique that simply asks the patient at the end of the visit ‘did you get everything you needed today?’ may lead to meaningful feedback and aligns well with the concept of using ‘did the patient get safe, effective, patient-centred’ as the primary frame of reference during direct observation [52]. While these two techniques might be of benefit, more research is needed before using patients and the inter-professional team for higher-stakes assessment. Research strongly suggests that multi-source feedback (representing the observations of an inter-professional group) should not be routinely used for higher-stakes assessment [163].

#### Guideline 33.

*Does direct observation influence learner and patient outcomes?*

Despite the central role of direct observation in medical education, few outcome data exist to demonstrate that direct observation improves learner and patient outcomes. Clinical and procedural competencies are foundational to safe, effective, patient-centred care. While evidence is lacking to show that direct observation assessments improve learner outcomes, and therefore patient outcomes, logic and indirect evidence do exist. Deliberate practice and coaching support skill improvement and the development of expertise [168]. The evidence that better communication skills among health professionals is associated with better patient outcomes strongly supports the importance of observing and providing feedback about such skills to ensure high levels of competence [169]. Conversely, as pointed out earlier, direct observation is infrequent across the continuum and there are gaps in practising physicians’ competencies [170–172]. Thus, it would be illogical to conclude direct observation is not important, but much more work is needed to determine the best methods that maximize the impact on learner and patient outcomes.

### Summary

We have compiled a list of guidelines focused on direct observation of clinical skills, a longstanding assessment strategy whose importance has heightened in the era of competency-based medical education. This work synthesizes a wide body of literature representing multiple viewpoints, was iterative, and represents our consensus of the current literature. Because this was not a systematic review, we may have missed studies that could inform the guidelines. Authors were from North America, potentially limiting generalizability of viewpoints and recommendations. Although we used group consensus to determine the strengths of each guideline, our interpretation of evidence strength was subjective.

## Conclusions

These guidelines are designed to help increase the amount and quality of direct observation in health professions education. Improving direct observation will require focus not just on the individual supervisors and their learners but also on the organizations and cultures in which they work and train. Much work remains to be done to identify strategies and interventions that motivate both supervisors and learners to engage in direct observation and that create a supportive educational system and culture in which direct observation (and the feedback that follows) is feasible, valued and effective. The design of these approaches should be informed by concepts such as self-regulated learning and the growing understanding of rater cognition. Designing, disseminating and evaluating such strategies will require an investment in educational leaders prepared to engage in the very difficult work of culture change. To our knowledge, such a multifaceted, comprehensive approach to improving direct observation of clinical skills by simultaneously focusing on educational leaders, supervisors, and learners, while considering the context, culture and system has not been described. Empowering learners and their supervisors to use direct observation to assess progress and inform higher-stakes assessments enables the educational system as a whole to improve learners’ capabilities and enhance the care of patients.
